# Selection of Natural Fibre for Pultruded Hybrid Synthetic/Natural Fibre Reinforced Polymer Composites Using Analytical Hierarchy Process for Structural Applications

**DOI:** 10.3390/polym14153178

**Published:** 2022-08-04

**Authors:** Thinesh Sharma Balakrishnan, Mohamed Thariq Hameed Sultan, Jesuarockiam Naveen, Farah Syazwani Shahar, Muhammad Imran Najeeb, Ain Umaira Md Shah, Tabrej Khan, Tamer Ali Sebaey

**Affiliations:** 1 Department of Aerospace Engineering, Faculty of Engineering, Universiti Putra Malaysia, Serdang 43400, Selangor Darul Ehsan, Malaysia; 2 Laboratory of Biocomposite Technology, Institute of Tropical Forestry and Forest Products (INTROP), Universiti Putra Malaysia, Serdang 43400, Selangor Darul Ehsan, Malaysia; 3 Aerospace Malaysia Innovation Centre (944751-A), Prime Minister’s Department, MIGHT Partnership Hub, Jalan Impact, Cyberjaya 63000, Selangor Darul Ehsan, Malaysia; 4 School of Mechanical Engineering, Vellore Institute of Technology, Vellore 632014, India; 5 Engineering Management Department, College of Engineering, Prince Sultan University, Riyadh 11586, Saudi Arabia; 6 Mechanical Design and Production Department, Faculty of Engineering, Zagazig University, Zagazig 44519, Sharkia, Egypt

**Keywords:** analytical hierarchy process, hybrid polymer composites, natural fibre, synthetic fibre

## Abstract

Application of synthetic fibres in composites has been raising environmental issues due to carbon emissions from the production site and reliability on non-renewable resources upon production. Hence, this research sets as a preliminary study to select suitable natural fibres to be hybridized with glass fibres for the development of sustainable and high-performance hybrid composites as potential alternative to conventional pultruded fibreglass composites in structural profile applications. In this study, analytical hierarchy process (AHP) was conducted to select the ideal natural fibre as reinforcement in the hybrid pultruded FRP composites suitable for structural applications. Hence, 13 natural fibre candidates were selected as alternatives and six criteria were chosen and analysed to select the best candidate for pultruded hybrid FRP. Criteria such as tensile strength, tensile modulus, density, cellulose content, elongation, and availability of fibres were assigned as the standard of selecting natural fibres for the application intended in this study. Among the 13 alternatives, kenaf was found to be the most suitable reinforcement for the application as it yielded the highest priority vector at 0.1. The results were then validated by carrying out sensitivity analysis to ensure kenaf is the most suitable material for the research.

## 1. Introduction

Every year, composites continue to replace traditional materials such as steel, aluminium, metals, and alloys implemented in structural applications in various industries. This is due to the superior properties composite materials can offer such as high strength-to-weight ratio, high durability, and wide design options. Composites can be manufactured using various manufacturing techniques depending on the resin type, reinforcement type, fibre placement, and process [1]. The popular manufacturing techniques such as pultrusion, resin transfer moulding, filament winding, and compression moulding are being widely used in composite industries [2]. Among these techniques, pultrusion is gaining significant attention from various industries due to its ability to produce structural FRP profiles with a high fibre volume and excellent strength.

Pultrusion is known for efficiently producing fibre reinforced composites with constant cross-sections such as flat bars, beams, channels, rods, and solid and hollow sections utilizing reinforcing fibres, resin, catalyst, fillers, pigments, and release agent. The automated process is designed for manufacturing linear constant cross-section, consistently and endlessly, and then reliably cutting them into pre-programmed lengths [3]. The closed moulding process does not only limit its scope in producing open-section geometries but also single- or multi-celled close-shaped profiles are possible to be made via pultrusion [4,5]. Pultrusion process is gaining more attention in manufacturing industries due to its continuous production and low labour cost [1]. Pultrusion process is commonly used to produce composites with synthetic fibre reinforcements such as glass and carbon fibres. However, these fibres are made from petroleum-based resources. Hence, to reduce the need of depending on non-renewable resources for structural products, researchers are developing bio-composites made from natural fibre reinforcement which is sustainable and eco-friendly.

Natural fibre composites offer some specific advantages over synthetic fibres such as lighter weight, lower cost, recyclable, and environmentally friendly. However, although natural fibres have advantages in composite production, some major drawbacks, such as their low impact strength, low thermal stability, and high moisture absorption properties, effects their long-term service behaviour and limits their use in extreme outdoor applications [6,7]. The high moisture absorption behaviour in natural fibre composites is due to the hydrophilic nature of natural fibres [8]. This may result in swelling of fibres, eventually reducing the mechanical strength and dimensional stability of the composites. Alomayri et al. [9] stated that the moisture absorption in cotton fabric composites resulted in reduction of flexural, impact, hardness, and fracture toughness properties.

Alternatively, natural fibres are combined with synthetic fibres to enhance the mechanical performance and water-resistant properties of composites. Extensive studies have been carried out on natural fibre reinforced hybrid composites covering investigations on long- and short-term properties to identify suitable application areas of the composites. Antigoni Barouni et al. [10] evaluated the fatigue properties of hybridized flax and glass composites by comparing them with flax reinforced composites. Their findings indicated that alternating layers of flax/glass hybrid composites show better fatigue properties with good fatigue life which are suggested for semi-structural applications. Similarly, research on fatigue life cycle of jute/glass reinforced hybrid composites for axial flow fan blades recorded 78% of fatigue life when compared to conventional glass fibre reinforced composites [11]. The researchers suggested that the hybrid composites can be applicable in fan blades at low normalised peak stresses. According to Kin Liao et al. [12], the durability of bamboo fiber reinforced polypropylene is enhanced when hybridized with glass fibers. The environmental aging studies show that the strength degradation of hybrid samples resulted in nearly two times less than that of bamboo fibre reinforced composites. Additionally, Srinivas et al. [6] stated that the mechanical properties of the natural/synthetic fibre reinforced hybrid composites improved with increasing volume fraction of glass fibres until a certain point where the negative hybrid effect takes place due to the formation of agglomerates.

Although hybridization can be a solution for environmentally friendly and cost-effective structural members, the individual fibre constitutes possess some drawbacks that narrows down their application in specific areas. For instance, glass fibres are very fragile to the alkaline environment of concrete due to some possible chemical reactions and may succumb to unsightly color fading due to exposure to UV radiations, while natural fibers with high-water absorption limits their application range in extreme environmental conditions. Abassi et al. [13] studied the durability of GFRP bars under temperature and alkaline environment. The study revealed that the properties of GFRP rebar deteriorate with temperature and time of exposure to alkaline environments. Amaro et al. [14] investigated the flexural and impact properties of glass fibre reinforced epoxy composites in alkaline and acidic environment. The study indicated that the properties of the composites degraded overtime in both conditions where alkaline environment contributes to more strength degradation compared to acidic environment. However, hybridizing glass fibres with natural fibres in composites have reduced the limitations which effect the durability of the materials. To analyse the hybridization effects due to environmental conditions, studies on hydrothermal ageing of jute/glass reinforced hybrid composites have been carried out by Phani et al. [15]. The studies indicated that the strength readings of hybrid and pure composites are closer to each other for long-term aging. Interestingly, the research also indicated that hybridizing jute with glass fibres reduced the rate of degradation over that of glass fibre composites. Akhila et al. [16] studied the mechanical performance and durability of hybrid sisal/glass fibre reinforced polymer composites for retrofitting of reinforced concrete structures by comparing them with glass fibre, sisal fibre, and carbon fibre reinforced polymer composites, respectively. All the samples were prepared where the FRPs were used as external confinement of concrete cylinders. The studies indicated that the performance of hybrid sisal/glass is higher compared to individual sisal and glass fibre composites in terms of axial load carrying capacity, ductility, and energy absorption rate. The researchers also suggested that the hybrid composites potentially could replace carbon fibres in concrete retrofitting applications. According to Mayandi et al. [17], the durability and mechanical performance could be improved when synthetic fibres such as glass fibres are used in outer layers of hybrid composites. This is because the UV resistance, alkali resistance, and moisture absorption properties are found to be better in glass fibres compared to natural fibres, hence protecting the inner layers of natural fibres from extreme environmental effects.

Overall, studies have shown that the hybridization of composites is known to be one of the approaches to overcome the limitations of the natural and synthetic materials by combining both materials to utilize and upgrade the unique aspects of the individual material [18,19]. The combination of materials is more suitable for light-load structural applications where a short life of the product is advantageous [6]. Natural/glass fibre reinforced hybrid composites can be applied into various light-load applications such as ladders, window frames, door panels, biomedical equipment, sports equipment, and automotive and aerospace interior applications [20,21,22,23]. Hence, being a good potential reinforcement replacing synthetic fibres in fibre reinforced composites, natural fibres can be hybridized with glass fibres via pultrusion for the development of pultruded FRP profiles for load bearing structural applications with respect to their mechanical and physical performances. However, before getting into other stages of research such as composites characterization, structural analyses, and product development, it is important to find the ideal fibre as reinforcement in pultruded FRP profile applications. Hence, the aim of this study is to use analytical hierarchy process (AHP) as a tool to find the ideal natural fibre for the pultrusion application as a preliminary action.

AHP is a multiple-criteria decision-making (MCDM) analysis technique used in solving decision-making problems by systematic and quantitative selection approach [24,25]. Researchers have been using AHP analysis as a problem-solving tool for making decisions intended in various applications. Dweiri et al. [26] conducted AHP analysis to select the suitable material for ‘keys’ using Expert Choice software. The analysis showed that high carbon steel scored the highest priority vector, hence the most suitable material for keys. Naveen et al. [27] produced an armour which was the hybridization between Kevlar 29 and the selected Cocos nucifera sheath from AHP method as the suitable natural fibre material. Fourteen natural fibres as alternatives and seven criteria were analysed in the study, where Cocos nucifera sheath scored the highest priority vector compared to other alternatives. Sapuan et al. [28] used AHP analysis to identify kenaf 60% reinforced polypropylene as the best choice for automotive dashboard panel application by considering mechanical and physical properties as the criteria with tensile strength, young’s modulus, and density as the sub-criteria. Besides serving as a simple and time saving tool, other advantages of AHP include it enables the decision maker to make consistent judgments by checking on the inconsistency and analysing the integrity of the final decision through sensitivity analysis [29,30,31,32].

In the current study, the comparison and selection of the most suitable natural fibre to be hybridized with glass fibre for pultrusion application was done by employing the AHP method. An AHP software, “Expert Choice 11.5”, was used to determine the optimal natural fibre for pultruded hybrid FRP bio-composites. Six criteria such as tensile strength, young’s modulus, density, elongation at break, cellulose content, and local availability were set for selection. For the material comparison, 13 natural fibres were considered as alternatives. The six criteria were formulated in the pairwise comparison matrix (PCM) with respect to the main goal. Then, the PCM of 13 alternative materials were formed with respect to the six criteria. By synthesizing the PCM data, the ranking of the proposed materials with respect to the different criteria, and the overall ranking with respect to the goal was obtained. The consistency ratio was made sure to be less than 0.10 (10%) to validate the judgments made. Finally, the simulation of results was obtained by using sensitivity analysis while also varying the weightage of each criterion.

Although there are a few works reported on natural/glass fibre reinforced composites, the real-life applications of natural/ synthetic fibre hybrid composites are relatively low and still under research and development. Therefore, due to the rising awareness of environmental issues, the main goal of this study is to find a suitable natural fibre material (among 13 alternatives) to be hybridized with glass fibres in developing environmentally friendly hybrid FRP composites for pultruded profile structural applications. Hence, the amount of glass fibres used in conventional pultruded products was reduced and the reliability on non-renewable resources to produce structural products was greatly cut down. Besides that, there is a huge gap needed to be filled by analysing the mechanical and physical properties covering the long-term and short-term behaviour of natural/glass fibre reinforced hybrid pultruded composites to identify its potential applications in various industries. Future studies will be carried out on the selected natural fibre hybrid composites to further analyse for its mechanical, physical, and structural performance and identify suitable application areas of bio-based pultruded structures before applying them into real life engineering applications and product development. Overall, the outcome of this preliminary study aims to serve as an initial stage of material selection and introducing the potential prospects of natural fibres into real life structural applications via bio-based pultruded profiles.

## 2. Design Framework and Criteria

### 2.1. Pultruded FRP Composites for Structural Application

Among the various types of composite manufacturing processes, pultrusion is one of the widely used closed moulding processing techniques to produce FRP profiles with high fibre volume and excellent strength [1,33,34,35]. Pultrusion is a continuous moulding process commonly used in many manufacturing industries to fabricate composite materials and structures of uniform cross-section.

By replacing conventional metals and alloys, pultruded FRP components are used in designing and building simple to complex structures for a wide range of applications. Pultrusion is chosen by many industries to manufacture parts that are strong, lightweight, and continuous which include the aerospace, marine, military, electrical, transportation, as well as infrastructure, chemical, and construction sectors. The profiles can be fabricated in any desired length with uniform cross-section. Besides that, pultruded products can also be cut, painted, drilled, and connected using screws, rivets, bolts, and adhesives which make them more user-friendly. Surface veils and UV-resistant coatings are typically applied to profiles which protect them from harmful radiations and corrosive environmental effects [36,37].

Typically, synthetic fibres such as glass fibres are widely used in manufacturing pultruded structures and profiles which are applied in multiple industries [38]. However, excessive use of synthetic materials in composites have been leaving some negative environmental impacts and awakening sustainability issues. This is due to the use of petrochemicals and dependency on non-renewable energy resources. Besides that, excessive use of synthetic fibre is also harmful to health, and it takes a long time to decompose its constituents. Hence, in this era where green technology is emerging vastly, natural fibres are proposed to be a superior alternative to synthetic materials in developing green composites for structural applications. With the renewed push towards environmental awareness we are currently experiencing, studies and research are being conducted by applying natural fibres into pultruded FRP composites to develop sustainable eco-friendly materials for structural applications.

Popular natural fibres such as kenaf, jute, flax, and hemp have been extensively researched in pultruded FRP composites [39,40,41,42,43,44]. K. Z. Zakaria et al., Hazizan Md. Akil et al., and F.H.A. Malek et al. have studied the mechanical properties of pultruded hybrid glass/natural fibre reinforced composites. Hybridised composites have shown better flexural strength and flexural modulus, according to K. Z. Zakaria et al. [45] and Hazizan Md. Akil et al. [46], compared to pure pultruded NFCs. According to F.H.A Malek et al. [47], pultruded hybrid glass/kenaf reinforced composites have outperformed pure pultruded NFC and GFC by recording higher bending strength and bending modulus at ratios of 60:40 and 80:20, respectively. Previous studies have proven that pultrusion process is suitable for developing natural/glass fibre reinforced hybrid composites. However, there is still a huge gap present in the scope of developing pultruded structural products using natural fibres as reinforcement. For instance, characterization using different types and forms of natural fibres (woven and yarns), analysis on stacking sequences, structural analyses on various types of cross sections of pultruded profiles, and product development is remaining as a gap to make bio-based pultrudes applicable in practical real-life applications. Hence, this preliminary study is intended to find the most suitable natural fibre to be hybridized with glass fibre to fill the prior gap mentioned and develop pultruded structural end-products with promising mechanical and physical properties.

Overall, hybridizing glass fibres with natural fibres through pultrusion process can be used in developing high performance eco-friendly structural parts. Natural fibres are not only chosen to solve sustainability issues; other properties such as light weight, lower cost, and excellent strength-to-weight ratio of these biomaterials make them popular in composite manufacturing.

### 2.2. Criteria in the Selection of Natural Fibre for Pultruded Structural Application

Setting the criteria plays a big role in the selection of natural fibres. Generally, the primary design factors to be considered to develop pultruded glass/natural fibre composites in structural applications are mechanical performance, weight, sustainability, and availability. In this study, the mechanical properties, physical properties, chemical properties, and availability were considered in setting the criteria. Table 1 shows the mechanical, physical, and chemical properties of natural fibres used in the AHP analysis.

#### 2.2.1. Tensile Strength

Tensile strength is the maximum load a material can withstand before failure. It is one of the basic requirements in developing structural components to withstand the forces applied before breaking under tension. Fibre strength is associated with improved tensile properties in the yarns and hence the composite [48]. Moreover, tensile properties are also important to avoid fibre breakage during pultrusion processing which causes defects in the finished product. This is due to the higher pulling force exerted on the pulling fibres during pultrusion processing. Therefore, it is important to select a fibre with higher tensile strength to avoid any failure during the pultrusion process and structural applications. Natural fibres have recorded an acceptable tensile strength to be applied in composites, although glass fibres recorded a slightly higher tensile strength of around 3400 MPa. Hence, hybridizing glass fibres with moderately high strength natural fibres are expected to produce sustainable, cost, and weight saving composites without sacrificing much tensile properties when compared to conventional pultruded fibreglass composites. The maximum intensity scale value was given to fibres with high tensile strength and the least scale was given to fibres with low tensile strength. Flax, kenaf, pineapple leaf, jute, and hemp fibres offer good tensile properties and hence were given a high intensity scale value.

#### 2.2.2. Tensile Modulus

Tensile modulus, which is also known as young’s modulus, is a measure of elasticity. Tensile modulus is the ratio of stress-to-strain applied along the longitudinal axis of the material. In that context, materials with high tensile modulus are stiffer than materials with low modulus. Fibres with higher modulus ensure good load transfer between the fibre and matrix, hence improving the modulus of the composite [49]. In this study, reinforcements with high tensile modulus were preferred to ensure a rigid composite for structural members. Flax, hemp, kenaf, and pineapple leaf fibres have high tensile modulus and hence were given the maximum intensity scale value.

#### 2.2.3. Density

Natural fibres typically have a lower density, hence resulting in lower weight composites when compared to synthetic fibre in reinforced composites. Another criterion in the fibre selection is the fibre density as it is directly related to the weight of structures, whereby the fibre’s density highly affects the ratio of strength-to-weight of the composites. The hybridization of glass/natural fibre reinforced composites has led to better mechanical properties and higher strength-to-weight ratio. Fibres with lower density are preferred to reduce the overall weight of the composites, thereby achieving structural components with light weight. Hence, higher priority vector after tensile properties was given to the density criteria with respect to the goal as hybrid glass and natural fibre in reinforced composites provide significant effects on the structural weight of composites when compared to conventional synthetic fibre reinforced composites. A maximum relative intensity scale value was given to fibres with lower density while a minimum scale was set for fibres with higher density.

#### 2.2.4. Elongation at Break

Elongation at break is used to determine the ductility of the material. Hence, the higher the elongation at break percentage, the higher the ductility of composites will be. High ductility composites will be more likely to deform and not break, whereas low ductility makes it brittle and fractures before deforming much under a tensile load [50]. Materials with good elongation properties aid in dissipating the kinetic energy of the structure upon plastic deformation. Therefore, by absorbing the impact before breaking, ductile materials reduce the safety risks [51]. Additionally, fibres with a high degree of elongation produce fabrics with superior properties such as tensile, tearing strengths, and flex resistance [48]. Generally, synthetic fibres such as glass fibres show better mechanical and physical properties compared to natural fibres. However, the specific modulus and elongation at break are better in natural fibres than the synthetic fibres [52]. These advantages of natural fibre properties serve as an important factor in developing natural/synthetic fibre reinforced hybrid composites which eliminates the weakest aspects from both natural and synthetic fibres while producing environmentally friendly materials with superior properties than conventional materials in structural applications. Alternatives with higher elongation were focused on in the analysis by setting them at a higher relative intensity scale.

#### 2.2.5. Cellulose Content

The chemical composition of natural fibre typically consists of cellulose, hemicelluloses, and lignin [53]. Cellulose is one of the main components in plant fibres present in the form of cellulosic micro fibrils in the inner layer of primary wall. Cellulose is crystalline with good strength, whereas hemicelluloses have random, amorphous structure with lower strength. Cellulose is made up of a linear chain of β-1, 4 linked D-glucose units which serves as the main element in the plant’s cell walls by providing a stiff and rigid structure to the plants. Generally, the reinforcing efficiency of cellulose fibres in natural fibre composites depends on the cellulose content and their crystallinity. A high cellulose content and low fibrillary angle improve the tensile properties in composites [54]. Cellulose chains link to form micro fibrils, in which the multiple hydroxyl (OH-) groups on the glucose residues hydrogen bond with each other, holding the chains firmly together and contributing to their high tensile strength [55]. Typically, crystallization in cellulose can reach up to 50–60% [56]. The crystallized structure of cellulose can result in better mechanical properties, such as a modulus of elasticity at approximately 150 GPa. Therefore, fibres with higher cellulose content are desirable to the application and have been rewarded a maximum intensity scale compared to fibres with lower cellulose content.

#### 2.2.6. Local Availability

The availability of natural fibres locally in Malaysia was also considered in the AHP analysis. Availability plays a vital role in developing structural parts and products so that the raw material supply is sufficient during production. Kenaf fibres and pineapple leaf fibres are widely available locally and hence given the most priority scale compared to other fibres. However, a minimum intensity scale value was given to ‘local availability’ with respect to the goal compared to other criteria included in this study. Technical criteria (tensile strength, tensile modulus, density, elongation, and cellulose content) were given more weightage than availability.

**Table 1:** Mechanical, physical, and chemical properties of fibres.

Fibres	Tensile Strength (MPa)	Tensile Modulus (GPa)	Density (g/cm^3^)	Elongation (%)	Cellulose Content (%)	References
Bagasse	20–290	19.7–27.1	1.2	1.1	52.4	[57,58]
Bamboo	230–295	17	1.1	11	54.6	[58,59,60,61]
Banana	355	33.8	1.35	5.3	65	[57]
Coir	220	6	1.25	15–25	43	[57]
Cotton	400	12	1.51	3–10	82.7	[57]
Flax	500–1500	60–80	1.4	1.2–1.6	64.1	[57]
Hemp	550–900	70	1.48	1.6	74.4	[57]
Jute	400–800	10–30	1.46	1.8	64.4	[57]
Kenaf	930	53	1.2	2.7–6.9	53.4	[57]
Oil Palm	248	3.2	1.55	2.5	65	[57]
Pineapple leaf (PALF)	170–1627	53	1.5	1–3	70	[57,62]
Ramie	500	44	1.5	2	68.6	[57]
Sisal	600–700	38	1.33	2–3	65.8	[57]
E-glass	2000–3500	70	2.5	2.8	-	[63]

## 3. AHP Methodology

**Figure 1:**
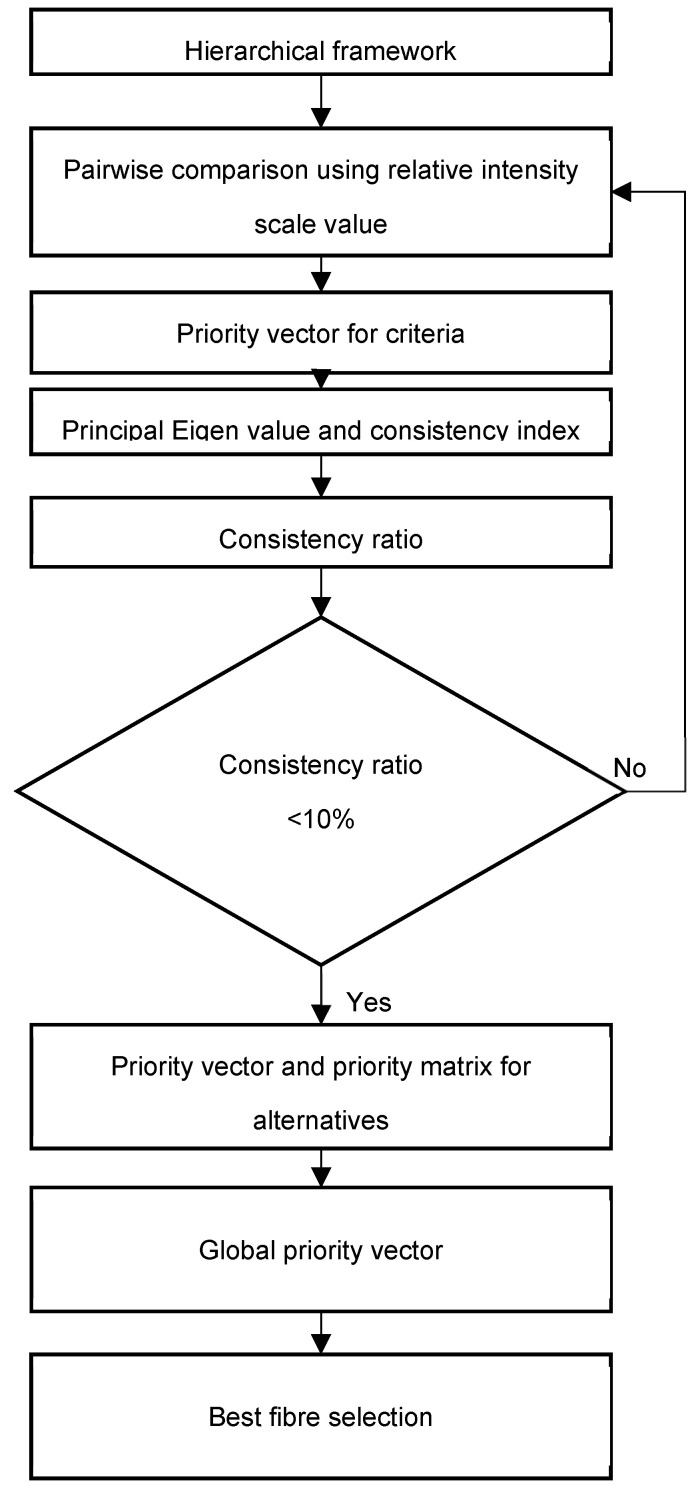
AHP methodology flowchart.

Typically, there are several stages in carrying out an analytical hierarchy process [63,64]. The steps in an analytical hierarchy process are demonstrated in a flowchart (Figure 1).

### 3.1. Hierarchical Structure

**Figure 2:**
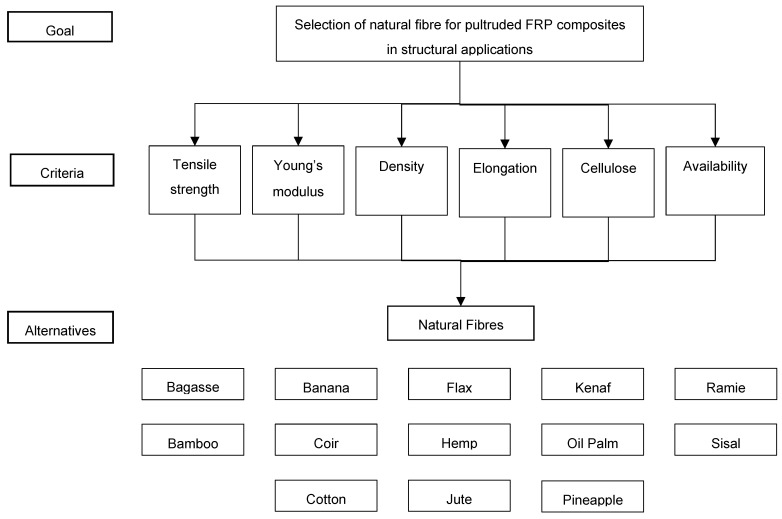
Hierarchical structure.

A hierarchical structure was developed to define the goal, criteria, and alternatives. The hierarchical structure of the AHP methodology is presented in Figure 2. The first level shows the overall goal for analytical hierarchy process. Based on the requirements to develop pultruded FRP profiles, six criteria were determined as the selection parameters, as shown in the second level of the hierarchical structure. The third level lists the natural fibres (alternatives) considered as candidate materials for hybridization with glass fibre for pultruded composites in structural applications.

### 3.2. Pairwise Comparison Using Relative Intensity Scale Value

Secondly, a pair-wise comparison matrix (PCM) was constructed for the criteria with respect to the goal using relative intensity scale. The intensity scale in Table 2 was used to indicate and compare the importance of one criterion in relation to another [65]. The intensity scale value varied from 1 to 9 to indicate the level of importance among the criteria.

**Calculation of Eigen vectors**

The PCM data from Step 2 was used to determine the relative priority vector of each attribute. The priority vector, *w*, was calculated as follows:

(1) $$\mathrm{Priority}\;\mathrm{vector},\;w\;=\frac{1}{n}\sum_{j=1}^{n}\frac{a_{ij}}{\sum_{i=1}^{a}a_{ij}},\;i,\;j\;=\;1,\;2,\;\text{…},\;n$$

where *a_ij_* is the importance scale (i.e., 1, 3, 5, …) and *n* is the number of criteria. 

### 3.3. Consistency Ratio

In the third stage, judgments consistency was validated using consistency ratio (CR). The consistency ratio was made sure to be less than 0.1 to ensure the judgments made were consistent and valid. However, the judgments are unreliable if the consistency ratio was more than 0.1. Hence, as shown in the flowchart in Figure 1, if the consistency ratio was more than 0.1, the PCM was required to be performed again until a ratio of less than 0.1 was obtained. The consistency ratio was calculated using the following equations.

**Calculation of Principal Eigen Value (λmax)**

(2) $$\mathrm{Principal}\;\mathrm{Eigen}\;\mathrm{value},\;\lambda \max\;=\sum_{i=1}^{n}\frac{\sum_{j=1}^{n}a_{ij}\;w_{j}}{w_{i}}\;\;i,\;j\;=\;1,\;2,\;\text{…},\;n$$

where *w* is Eigen vector, *a_ij_* is the importance scale (i.e., 1, 3, 5, …), and *n* is the number of criteria.

**Calculation of Consistency index**

Consistency Index, CI = (λ_max_ – *n*)/(*n* – 1) (3)

where *n* is the number of criteria.

**Calculation of Consistency ratio**

Consistency ratio, CR = CI/RI(4)

where CI is the consistency index and RI is the random consistency index as in Table 3.

### 3.4. Formulating and Synthesizing PCM of the Proposed Materials with Respect to Criteria

PCM for the alternative materials with respect to all the criteria were formulated in the fourth stage. Then, the alternative materials priority vector, (*w*), with respect to each criterion was calculated. The PCMs for the alternative materials with respect to the criteria were formulated using the data from previous research works on mechanical, physical, and chemical properties of each type of natural fibre illustrated in Table 1.

### 3.5. Identifying the Best Alternative According to Global Prioritization

As the final step, the global priority vector was generated by multiplying PCM for the alternative materials and the criteria’s priority vector. The ranking of global priority vector obtained from this test determines the most suitable proposed material [67]. The alternative material that yielded the highest priority vector is selected as the most suitable natural fibre to be implemented in the development of pultruded hybrid FRP composites.

## 4. Pairwise Comparison Matrix (PCM) Using AHP Software

**Figure 3:**
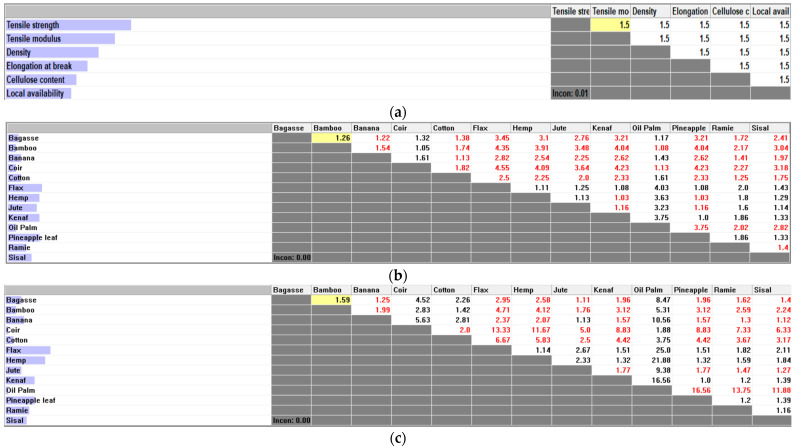

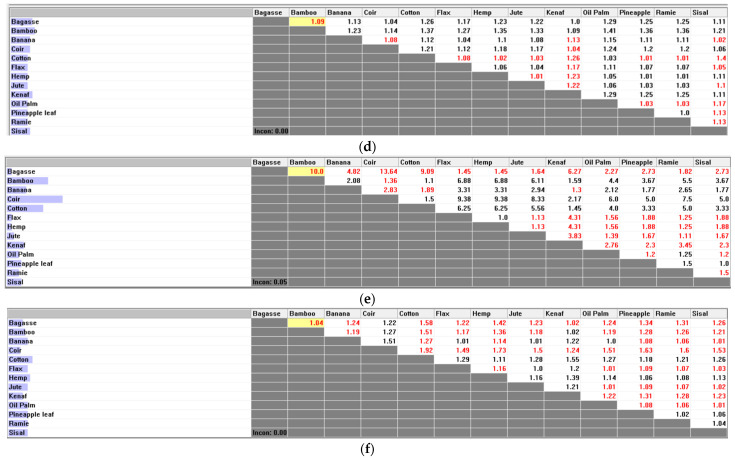
(**a**) Relative importance with respect to the goal. (**b**) Relative importance with respect to the tensile strength. (**c**) Relative importance with respect to the tensile modulus. (**d**) Relative importance with respect to the density. (**e**) Relative importance with respect to the elongation. (**f**) Relative importance with respect to the cellulose content.

Expert Choice v.11.5 software was used to formulate the PCM to identify the relative importance of criteria with respect to the goal and alternatives with respect to the criteria. Black coloured values indicate that the row member is relatively given higher priority than the column number, whereas red coloured values indicate that higher priority is given to the column member than the row member. Figure 3a shows the PCM of the criteria versus the goal. The criteria were ranked according to the following order: tensile strength > tensile modulus > density > elongation at break > cellulose content > availability. Higher weightage was given to the criteria with more importance in designing the pultruded composites for structural applications. In that context, tensile strength followed by tensile modulus were the most important factors considered to produce structural profiles with better strength and good stiffness, whereas density was considered third in the ranking to produce structural products with lower weight. Elongation at break which came after density aims to ensure good ductility, hence improving the safety risks upon failure. The PCM of alternatives with respect to each criterion is generated using the data collected on the parameters of each fibre, respectively, as listed in Table 1. Generally, a range of values were collected from previous studies for each property of plant fibres due to the variations in the fibre quality affected by different plantation regions and processing methods. Hence, the most common values obtained from several previous research were considered as fixed values to generate the PCM in this study. Figure 3b,c shows the PCM of the alternative materials with respect to the tensile strength and tensile modulus criteria, respectively. The relative importance scale values were obtained by calculating the ratio between the tensile values of individual fibres. For instance, in Figure 3b, the tensile strength given for bagasse and bamboo were 290 Mpa and 230 Mpa, respectively. Hence, by calculating their ratio (290/230), the relative importance scale value of 1.26 was obtained. The scale is indicated in black showing that the row member (bagasse) was relatively given higher priority than the column number (bamboo). Similarly in Figure 3c, the tensile modulus of bagasse and bamboo were 27.1 Gpa and 17 Gpa, respectively, where the corresponding ratio (27.1/17) was 1.59. The obtained value is indicated in black showing that bagasse is 1.59 times better than bamboo for the intended application. According to Figure 3d, the corresponding ratio of 1.09 indicated in red shows that bamboo has lower density than bagasse at 1.1 and 1.2 g/cm^3^, respectively. Hence, the value 1.09 was obtained from the ratio of (1.2/1.1). The same rule was applied in obtaining the relative importance scale of fibres with respect to the elongation at break and cellulose content as shown in Figure 3e,f, respectively.

## 5. Results and Discussion

With the aid of “Expert Choice” v.11.5 software, AHP was carried out to select the most suitable natural fibre as reinforcement in the pultruded hybrid FRP composites for structural applications. As the first step, PCM of the six criteria was made followed by PCM of the 13 alternatives with respect to the criteria.

**Figure 4:**
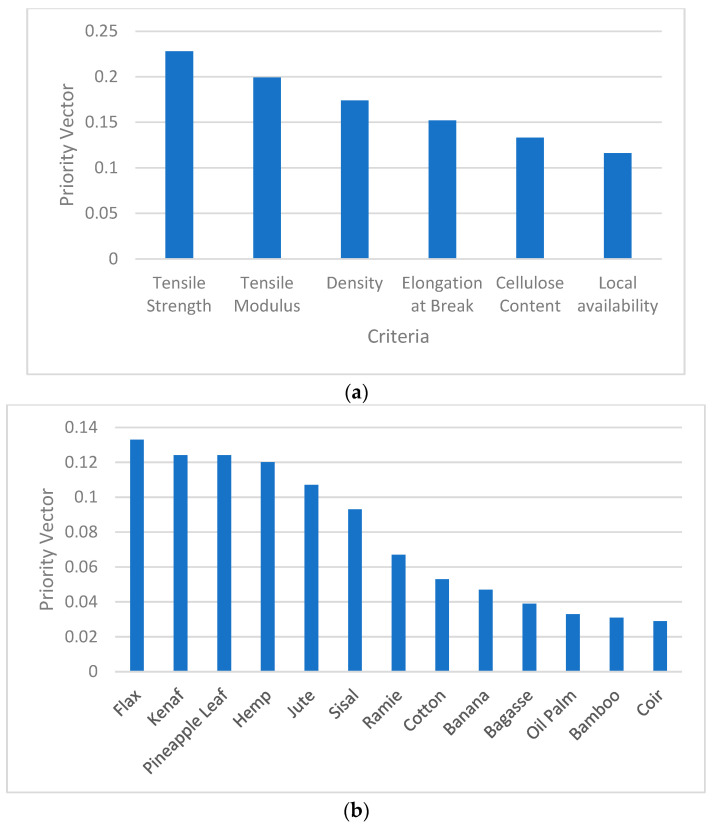

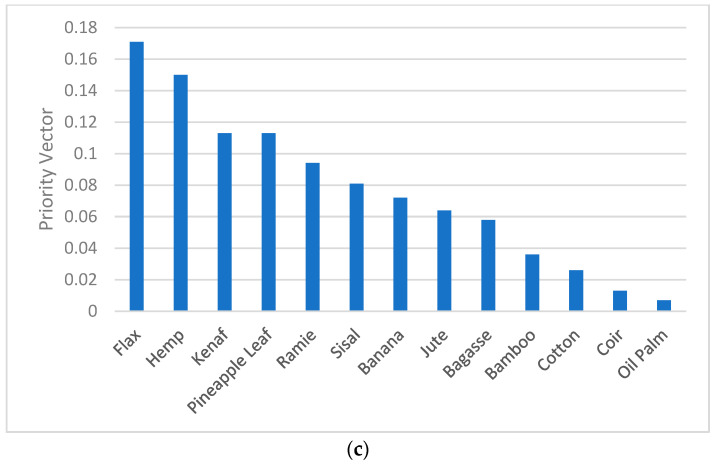
(**a**) Synthesis of criteria with respect to the goal. (**b**) Synthesis of natural fibres with respect to the tensile strength. (**c**) Synthesis of natural fibres with respect to the tensile modulus.

The criteria weightage was given in the following order: tensile strength > tensile modulus > density > elongation > cellulose > availability as shown in Figure 4a. According to the criteria ranking, the main factors in selecting the suitable natural fibre for structural applications is tensile strength, tensile modulus, followed by density. This is because materials with higher tensile strength, tensile modulus, and lower density are preferable in making structural profiles to improve its strength-to-weight ratio and stiffness-to-weight ratio. It is also clear that the strength of the material plays a vital role when selecting natural fibres for structural applications. Hence, high tensile strength in fibres is given the most priority among other criteria to ensure the application of the fibres in load bearing applications.

Figure 4b shows the synthesis of the natural fibres with respect to the tensile strength. The alternatives were ranked in the following order: flax > kenaf > PALF > hemp > jute > sisal > ramie > cotton > banana > bagasse > oil palm > bamboo > coir. Figure 4c demonstrates the synthesis of candidate alternatives with respect to the tensile modulus where the ranking was ordered as follows: flax > hemp > kenaf > PALF > ramie > sisal > banana > jute > bagasse > bamboo > cotton > coir > oil palm. Based on the results obtained on tensile strength and tensile modulus, fibres such as flax, kenaf, pineapple leaf, and hemp have scored significantly higher priority vector which proves their potential in load bearing applications. Although the mentioned natural fibres have lower mechanical properties compared to glass fibres which are conventionally used in producing high load bearing structural profiles, the moderate mechanical property in natural fibres widens the opportunities to develop moderate load bearing structural applications by greatly cutting down the cost and reducing sustainability issues. Hence, structural products such as frames, ladders, aerospace and automotive interior elements, and sports equipment can be manufactured with good strength property via pultruded natural/glass fibre reinforced hybrid composites.

**Figure 5:**
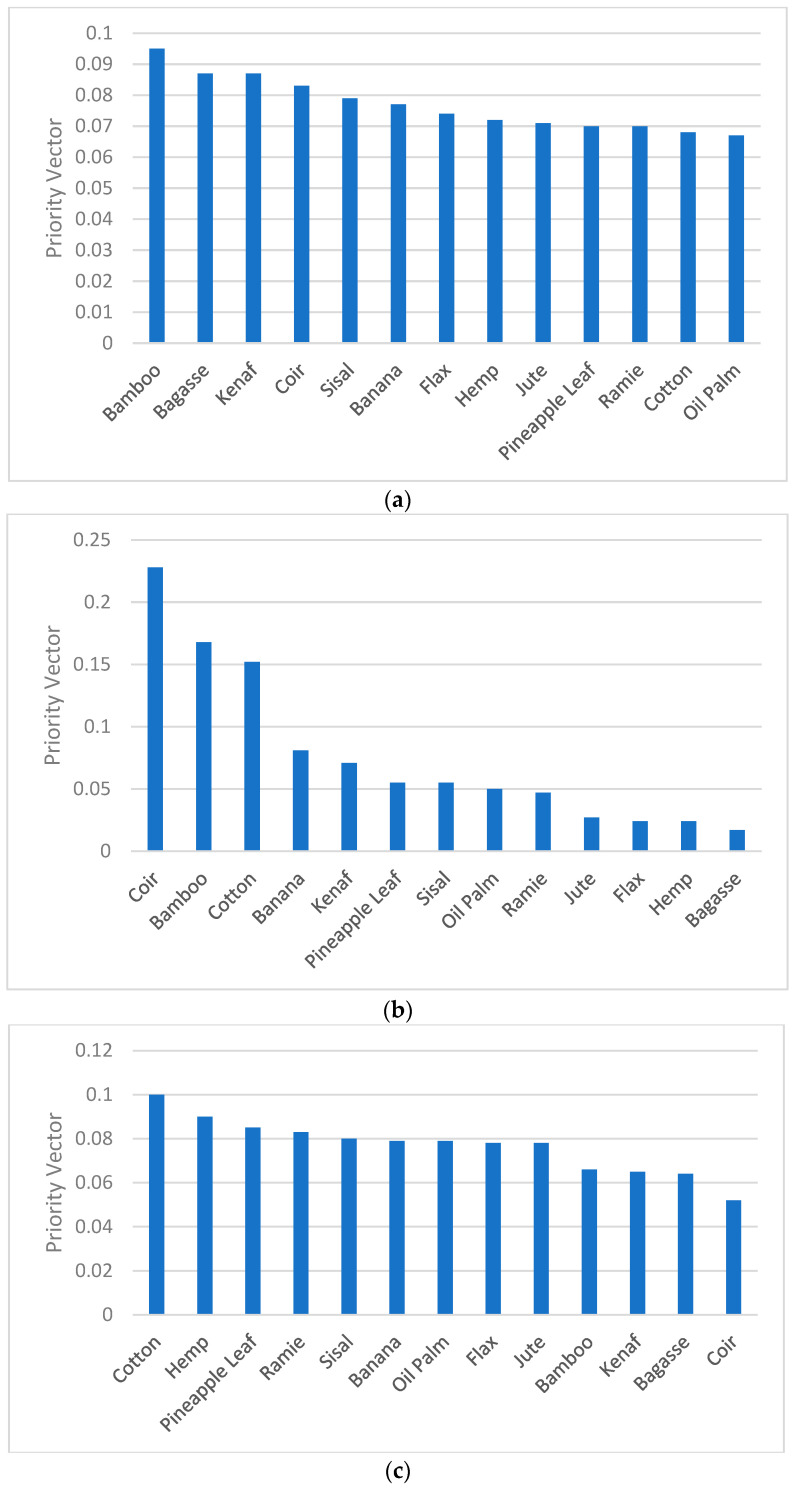
(**a**) Synthesis of natural fibres with respect to the density. (**b**) Synthesis of natural fibres with respect to the elongation at break. (**c**) Synthesis of natural fibres with respect to the cellulose content.

Figure 5a shows the synthesis of the alternatives with respect to the density where the fibres were ranked in the following order: bamboo > bagasse > kenaf > coir > sisal > banana > flax > hemp > jute > PALF > ramie > cotton > oil palm. The ranking highlights that values for priority vector scored by alternatives are close to each other and the differences are only at a minor level. Based on the ranking given, bamboo, bagasse, kenaf, and coir have lower density (ranging up to 0.08 to 0.09) than the average values shown by other candidate fibres (ranging about 0.07 to 0.08). The lower density in fibres is also important in developing profiles at much lower weight. However, by considering the tensile properties in Figure 4b,c, kenaf shows the potential to develop profiles with higher strength-to-weight ratio and stiffness-to-weight ratio compared to other candidates. Figure 5b shows the synthesis of the candidate fibres with respect to the elongation at break. The fibres ranking was in the following order: coir > bamboo > cotton > banana > kenaf > PALF > sisal > oil palm > ramie > jute > flax > hemp > bagasse. Fibres such as coir, bamboo, and cotton have shown significantly higher priority vector (more than 0.15), whereby banana, kenaf, pineapple leaf, and sisal scored moderate priority vector (more than 0.05) with respect to the elongation at break. These materials add ductility to the structural products by dissipating the kinetic energy of the structure upon plastic deformation. Hence, the safety risk is significantly improved. Figure 5c shows the synthesis of the alternatives with respect to the cellulose content. The ranking obtained from the results were in following order: cotton > hemp > PALF > ramie > sisal > banana > oil palm > flax > jute > bamboo > kenaf > bagasse > coir. Cellulose is crystalline which provides good strength and rigidity to the fibres. Almost all the alternatives demonstrated values for cellulose content near to each other. However, the highest cellulose content is present in cotton and the lowest cellulose content is present in coir fibres. Similarly, the synthesis of fibres with respect to availability was done and evaluated. To ensure a consistent supply to the production of materials, fibres which are made available locally were given higher priority scale compared to other alternatives. Among the fibres available in Malaysia include kenaf, pineapple leaf, bamboo, bagasse, coir, banana, and oil palm. Kenaf fibres and pineapple leaf fibres are widely available locally and hence given the most priority scale compared to other fibres.

**Figure 6:**
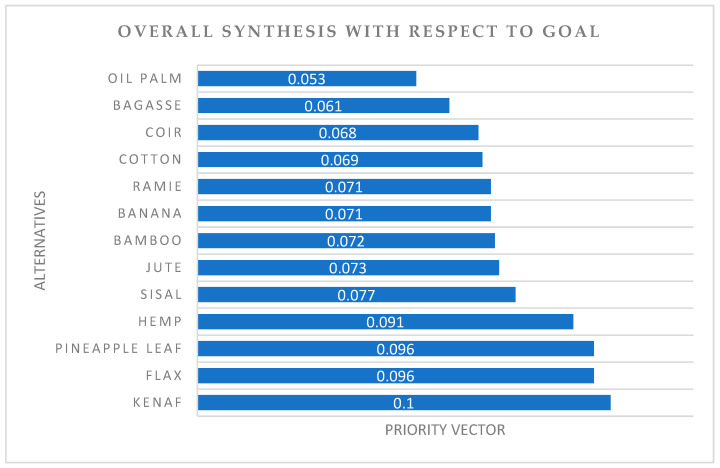
Overall synthesis result.

The priority vector of the alternative materials with respect to the criteria was obtained by synthesizing the PCM, which is shown in Figure 6. The figure shows that kenaf fibre is the most suitable alternative to be hybridized with glass fibre in pultruded FRP composites for structural application. The top four potential alternatives for light load bio-based pultruded profile application are listed in order as kenaf, flax, pineapple leaf, and hemp fibres. Kenaf scored the highest priority vector (0.1), followed by pineapple leaf (0.096), flax (0.096), and hemp (0.091). However, oil palm fibre has recorded the least value (0.053). This shows that oil palm fibre is least suitable to be chosen for hybrid composites in pultruded profiles. The overall consistency ratio recorded was 0.01, which proves that the judgements made during the AHP procedure were consistent and valid.

**Figure 7:**
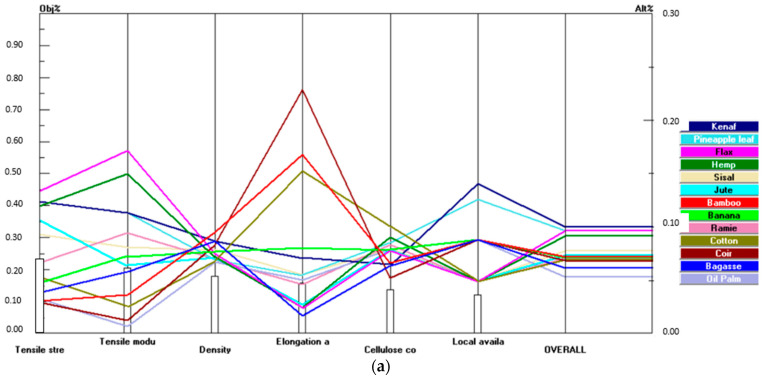

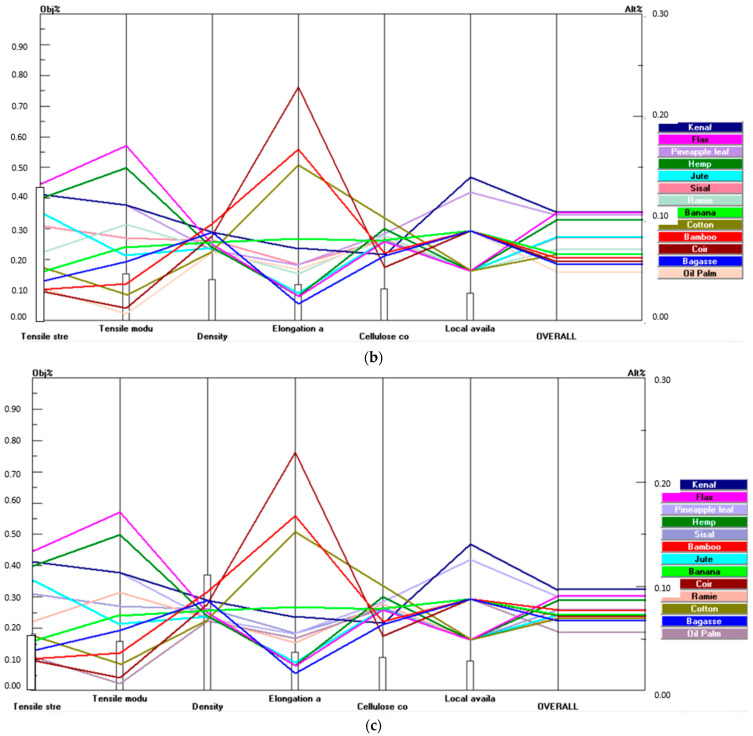
(**a**) Performance chart of the priority vector. (**b**) Performance chart with the priority vector increased to 20% for tensile strength. (**c**) Performance chart with the priority vector increased to 20% for density.

Sensitivity analysis was used to get the verification and validation of the overall results of the AHP process. Figure 7a shows the performance chart demonstrating the priority vector yielded by the natural fibres with respect to the criteria.

As the final step, the variation on the ranking scenarios of the natural fibres corresponding to each criterion was validated by increasing the priority vector of each criterion by 20% weightage. Figure 7b,c demonstrates the performance charts of the alternatives varied with respect to the tensile strength and density, respectively, when the priority vector was increased by 20%. Performance charts were obtained for other criteria as well and the obtained results are summarized in Table 4.

Sensitivity analysis is carried out to study the impact on the results when the weightage of the priority vector for each criterion is varied. Typically, the result of the analysis is considered robust when the changes are not significant due to variations in underlying assumptions. From the sensitivity analysis, kenaf fibre is ranked as the best alternative in four out of six criteria (tensile strength, density, cellulose, and availability). However, for tensile modulus and elongation, kenaf holds the third and second position, respectively, when compared to other alternatives. Therefore, the selection of kenaf as the suitable alternative using AHP is proven to be robust as consistent results were obtained on kenaf fibre when the weightage of priority vector in each criterion was altered. The sensitivity analysis validates the results obtained earlier by providing results on kenaf fibre as the most suitable fibre in the application of pultruded hybrid FRP composites for structural applications.

## 6. Conclusions

Based on the study, the following conclusions can be drawn:

1. Kenaf fibre is selected as the suitable alternative using AHP analysis, satisfying the basic design objectives and criteria of the application intended.
2. Consistency analysis was performed to validate the judgements made in the development of PCM. The overall inconsistency ratio obtained was 0.01, which is lower than the maximum CR value (0.1). Hence, it is proven that the judgements made during the AHP analysis were consistent and valid.
3. The sensitivity analysis shows that kenaf fibre is the best alternative as it scored the highest priority vector in four out of six criteria (tensile strength, density, cellulose, and availability). This further validates the fibre selection process using AHP.
4. Overall, the AHP method serves researchers and engineers as one of the effective multi-criteria decision-making tools to perform selection and problem-solving tasks systematically for various applications. There is still a huge scope of work to optimally select the suitable natural fibres for other various practical engineering applications where AHP can be used as a tool in various research areas. Additionally, pultruded kenaf/glass reinforced hybrid composites can be further analysed for its mechanical, physical, and structural performance to identify and validate its integrity in practical engineering structural applications.

## Figures and Tables

**Table 2:** Importance scale for PCM analysis [66].

Relative Intensity Scale	Definition
1	Equal importance of *i* and *j*
3	Slightly more importance of *i* over *j*
5	High importance of *i* over *j*
7	Very high importance of *i* over *j*
9	Extreme importance of *i* over *j*
2, 4, 6, 8	Intermediate values between two adjacent judgements

**Table 3:** Average Random Consistency [27].

Size of Matrix	1	2	3	4	5	6	7	8	9	10
RI	0	0	0.58	0.9	1.12	1.24	1.32	1.41	1.45	1.49

**Table 4:** Fibre ranking when priority vector increased by 20% for each criterion.

Rank	Tensile Strength	Tensile Modulus	Density	Elongation	Cellulose	Availability
1	Kenaf	Flax	Kenaf	Coir	Kenaf	Kenaf
2	Flax	Hemp	Flax	Kenaf	Palf	Palf
3	Palf	Kenaf	Palf	Bamboo	Flax	Flax
4	Hemp	Palf	Hemp	Cotton	Hemp	Hemp
5	Jute	Sisal	Sisal	Palf	Sisal	Bamboo
6	Sisal	Ramie	Bamboo	Flax	Jute	Banana
7	Ramie	Banana	Jute	Hemp	Cotton	Coir
8	Banana	Jute	Banana	Banana	Ramie	Sisal
9	Cotton	Bamboo	Coir	Sisal	Banana	Jute
10	Bamboo	Bagasse	Ramie	Ramie	Bamboo	Bagasse
11	Coir	Cotton	Cotton	Jute	Coir	Ramie
12	Bagasse	Coir	Bagasse	Oil Palm	Bagasse	Cotton
13	Oil Palm	Oil Palm	Oil Palm	Bagasse	Oil Palm	Oil Palm

## Data Availability

Not applicable.
